# Closing the Gaps in Care of Dyslipidemia: Revolutionizing Management with Digital Health and Innovative Care Models

**DOI:** 10.31083/j.rcm2412350

**Published:** 2023-12-13

**Authors:** Samuel J Apple, Rachel Clark, Jonathan Daich, Macarena Lopez Gonzalez, Robert J Ostfeld, Peter P Toth, Vera Bittner, Seth S Martin, Jamal S Rana, Khurram Nasir, Michael D. Shapiro, Salim S Virani, Leandro Slipczuk

**Affiliations:** ^1^Department of Medicine, New York City Health and Hospitals/Jacobi Medical Center, Albert Einstein College of Medicine, Bronx, NY 10461, USA; ^2^Division of Cardiology, Montefiore Medical Center/Albert Einstein College of Medicine, Bronx, NY 10467, USA; ^3^CGH Medical Center, Sterling, IL, and Division of Cardiology, Johns Hopkins University School of Medicine, Baltimore, MD 61081, USA; ^4^Division of Cardiovascular Disease, University of Alabama at Birmingham, Birmingham, AL 35294, USA; ^5^Digital Health Lab, Ciccarone Center for the Prevention of Cardiovascular Disease, Division of Cardiology, Johns Hopkins School of Medicine, Baltimore, MD 21287, USA; ^6^Division of Cardiology, The Permanente Medical Group, Kaiser Permanente, Oakland, CA 94611, USA; ^7^Division of Cardiovascular Prevention and Wellness, Houston Methodist DeBakey Heart & Vascular Surgery, Houston, TX 77030, USA; ^8^Center for Prevention of Cardiovascular Disease, Section on Cardiovascular Medicine, Wake Forest University School of Medicine, Winston-Salem, NC 27101, USA; ^9^Office of the Vice Provost (Research), The Aga Khan University, 74800 Karachi, Pakistan; ^10^Division of Cardiology, The Texas Heart Institute/Baylor College of Medicine, Houston, TX 77030, USA

**Keywords:** dyslipidemia, gaps in care, atherosclerotic cardiovascular disease, atherosclerosis, technology, telehealth, lipid-lowering therapy

## Abstract

Although great progress has been made in the diagnostic and treatment options for dyslipidemias, unawareness, underdiagnosis and undertreatment of these disorders remain a significant global health concern. Growth in digital applications and newer models of care provide novel tools to improve the management of chronic conditions such as dyslipidemia. In this review, we discuss the evolving landscape of lipid management in the 21st century, current treatment gaps and possible solutions through digital health and new models of care. Our discussion begins with the history and development of value-based care and the national establishment of quality metrics for various chronic conditions. These concepts on the level of healthcare policy not only inform reimbursements but also define the standard of care. Next, we consider the advances in atherosclerotic cardiovascular disease risk score calculators as well as evolving imaging modalities. The impact and growth of digital health, ranging from telehealth visits to online platforms and mobile applications, will also be explored. We then evaluate the ways in which machine learning and artificial intelligence-driven algorithms are being utilized to address gaps in lipid management. From an organizational perspective, we trace the redesign of medical practices to incorporate a multidisciplinary team model of care, recognizing that atherosclerotic cardiovascular disease risk is multifaceted and requires a comprehensive approach. Finally, we anticipate the future of dyslipidemia management, assessing the many ways in which atherosclerotic cardiovascular disease burden can be reduced on a population-wide scale.

## 1. Introduction

Atherosclerotic cardiovascular disease (ASCVD), encompassing coronary artery disease (CAD), stroke and peripheral artery disease, is the leading cause of death worldwide [1]. A large body of evidence has established that low-density lipoprotein and other apolipoprotein B (apoB)-containing lipoproteins are key modifiable risk factors with a causal role in ASCVD [2]. The current canonical view suggests that these atherogenic lipoproteins penetrate the endothelium and enter the arterial wall, inducing a maladaptive inflammatory process that leads to the initiation of atherogenesis. Atherosclerotic plaque gradually evolves and, as it becomes unstable, can rupture with formation of an overlying thrombus, culminating in an acute cardiovascular event [3, 4]. Accelerated by major risk factors, including smoking, hypertension, and diabetes, as well as emerging, nontraditional risk factors, such as pregnancy-related disorders, autoimmune disease and depression [5], apoB lipoproteins promote atherogenesis over the course of a lifetime [6, 7]. Thus, rather than viewing low-density lipoprotein cholesterol (LDL-C) as a static measure, many have recently advocated for a shift in perspective towards assessing an individual’s cumulative cholesterol exposure, or “cholesterol-years”, a framework akin to “pack-years” regarding tobacco exposure [8], and argue that screening and treatment of LDL-C should be started early and intensively [2, 9, 10].

Our objective in this review is to discuss the evolving landscape of lipid management in the 21st century, identify current treatment gaps and explore possible solutions through digital health and new models of care. After outlining the evidence base for lipid-lowering therapies (LLT) and areas for improvement, we examine the history and development of value-based care and the national establishment of quality metrics for various chronic conditions. These concepts on the level of healthcare policy not only inform reimbursements but also define the standard of care. Next, we consider the advances in clinical assessment of ASCVD risk score calculators as well as evolving imaging modalities. The growth and potential role of digital health, ranging from telehealth visits to online platforms, artificial intelligence-driven algorithms and mobile applications, will be explored. We also evaluate the ways in which machine learning and artificial intelligence-driven algorithms are being utilized to address gaps in lipid management. From an organizational perspective, we will trace the redesign of medical practices to incorporate a multidisciplinary team model of care, recognizing that ASCVD risk is multifaceted and requires a comprehensive approach. Lastly, we anticipate the future of lipid management, assessing the many ways in which ASCVD burden can be reduced on a population scale.

## 2. Benefits of Lipid-Lowering Therapies 

Since the discovery of statins in the mid-1970s [11], the past few decades of research have produced a growing arsenal of LLT. In addition to lifestyle modifications, initiation of LLT in qualifying patients for both primary and secondary prevention achieves significant protective effects against the development and progression of ASCVD [12, 13, 14, 15, 16].  To illustrate, a patient-level meta-analysis of 26 randomized controlled trials (RCTs), either comparing different statin doses or comparing statins to controls for primary or secondary prevention, including nearly 170,000 patients over a median follow-up time of 4.8 years, demonstrated that all-cause mortality was decreased by 10% per 1.0 mmol/L (38.6 mg/dL) reduction in LDL-C (relative risk [RR] 0.90, 95% confidence interval [CI] 0.87–0.93; *p* < 0.0001), largely reflecting significant reductions in deaths due to coronary heart disease (RR 0.80, 99% CI 0.74–0.87; *p* < 0.0001) [17]. Intensification of LLT for those not at goal on maximally tolerated statin therapy has demonstrated additive value. The Improved Reduction of Outcomes: Vytorin Efficacy International Trial (IMPROVE-IT) analyzed the effect of adding ezetimibe, an inhibitor of intestinal cholesterol absorption, to simvastatin in 18,144 patients hospitalized for acute coronary syndrome (ACS) within the preceding 10 days [16]. At 7 years, patients in the ezetimibe group had a decreased primary composite end point of cardiovascular death, major coronary events, or nonfatal stroke compared to the simvastatin-monotherapy group (32.7% vs 34.7%; hazard ratio [HR], 0.936; 95% CI, 0.89–0.99; *p* = 0.016). Likewise, in a landmark trial assessing the cardioprotective effects of the proprotein convertase subtilisin–kexin type 9 inhibitor (PCSK9-I) evolocumab, the Further Cardiovascular Outcomes Research with PCSK9 Inhibition in Subjects with Elevated Risk (FOURIER) trial randomized 27,564 high-risk patients with clinical ASCVD who were taking a regimen of statin +/- ezetimibe to either evolocumab or placebo [18]. Patients who received evolocumab had LDL-C levels lowered by 63% from baseline as compared with placebo after 12 weeks, from a median of 92 mg/dL (2.4 mmol/L) to 26 mg/dL (0.67 mmol/L). Additionally, evolocumab treatment reduced the risk of a composite of cardiovascular death, myocardial infarction (MI), stroke, hospitalization for unstable angina, or coronary revascularization (9.8% vs 11.3%; HR, 0.85; 95% CI, 0.79–0.92; *p* < 0.001). Lastly, the ODYSSEY OUTCOMES trial, which randomized the PCSK9-I alirocumab versus placebo in 18,924 patients who had ACS 1–12 months prior and were receiving a high-intensity or maximum tolerated statin dose and either LDL-C >70 mg/dL, non-high-density lipoprotein cholesterol (non-HDL-C) >100 mg/dL or apoB >80 mg/dL, illustrated that those in the PCSK9-I group had a lower rate of death from CAD, nonfatal MI, fatal or nonfatal ischemic stroke, or unstable angina requiring hospitalization compared to placebo (9.5% vs 11.1%, HR, 0.85; 95% CI 0.78–0.93; *p* < 0.001) [19].

Beyond statin initiation, current guidelines emphasize appropriate statin dose intensification, as well as addition of non-statin LLT when indicated, depending on each patient’s major risk factors (e.g., diabetes mellitus [DM], cigarette smoking, hypertension), risk enhancing factors (e.g., family history, metabolic syndrome, chronic kidney disease, chronic inflammatory disorders, preeclampsia or eclampsia), and response to therapy—in particular, relative and absolute reductions in LDL-C [20]. The 2022 American College of Cardiology (ACC) Expert Consensus Decision Pathway (ECDP) on the Role of Nonstatin Therapies for LDL-C Lowering in the Management of ASCVD Risk also note that for some patients with LDL-C ≥190 mg/dL (4.92 mmol/L) and additional risk factors for whom statin monotherapy is highly unlikely to sufficiently reduce LDL-C by 50% or to <100 mg/dL, co-initiation of both statin and non-statin LLT initially may be indicated for primary prevention [21].

For secondary prevention, the potential benefits of upfront combination LLT were recently described by Lewek *et al*. [22] in a propensity-matched retrospective analysis of 1536 post-ACS patients using the Polish Registry of Acute Coronary Syndromes. Their analysis found that upfront combination therapy was associated with a significant reduction of all-cause mortality in comparison with statin monotherapy (odds ratio [OR], 0.526 [95% CI, 0.378–0.733]), with absolute risk reduction of 4.7% after 3 years (number needed to treat [NNT] of 21). These findings may, in part, be explained by a reduction in the delay to therapeutic target achievement using combination therapy instead of a stepwise approach proceeding from statin monotherapy. Based on these data and similar reports [23, 24], some have suggested that upfront combination therapy may benefit all patients with known ASCVD (with few exceptions, such as in patients with limited life expectancy), much in the same way that guidelines for other chronic conditions, such as hypertension, diabetes and heart failure with reduced ejection fraction, advocate for upfront combination given the clear evidence for benefit using multiple agents [25]. Combining therapies also has the potential to decrease the prevalence of dose-dependent adverse events by allowing for lower doses of each respective agent, which may mitigate side effects attributed to statins. Lastly, advocates for upfront combination LLT additionally stress that combination therapy has a greater maximum capacity to lower LDL-C compared to monotherapy [26], likely due to the synergistic effect of targeting multiple pathways of lipid metabolism.

In addition to upfront combination therapy, single-pill combinations have been shown to significantly improve medication adherence, a frequent barrier to adequate LDL-C reduction. For example, a retrospective analysis of 311,242 outpatients at very-high cardiovascular risk treated by general practitioners and cardiologists in Germany between 2013 and 2018 demonstrated that patients who received a combination pill had significantly greater reductions in LDL-C [reduction 28.4% (40.0 ± 39.1 mg/dL)] as compared to those receiving the exact same medications as separate pills [19.4% (27.5 ± 33.8 mg/dL)]; *p* < 0.0001 [27]. Furthermore, the Use of a Multidrug Pill in Reducing Cardiovascular Events (UMPIRE) trial randomized 2004 participants with established cardiovascular disease or estimated 5-year cardiovascular risk of over 15% were randomized to polypill-based treatment (aspirin 75 mg, simvastatin 40 mg, lisinopril 10 mg and either atenolol 50 mg or hydrochlorothiazide 12.5 mg) versus usual care [28]. Patients receiving fixed-dose combinations were found to have improved adherence compared to usual care (86% vs 65%; RR of being adherent, 1.33; 95% CI, 1.26–1.41; *p* < 0.001) with a concurrent reductions in LDL-C (–4.2 mg/dL; 95% CI, –6.6 to –1.9 mg/dL; *p* < 0.001) at the end of the study (with a median follow-up time of as 15 months). Beyond improving adherence and ASCVD outcomes in developed countries alone, the advent of polypills also carries the potential to bring effective ASCVD prevention within economic reach of individuals and governments of poorer countries [29].

Within secondary prevention patients, for a subgroup considered to have very high ASCVD risk, defined as a history of multiple major ASCVD events or 1 major ASCVD event and multiple high-risk conditions, the 2018 ACC and American Heart Association (AHA) multisociety guidelines recommended the addition of ezetimibe when the LDL-C level remains ≥70 mg/dL (≥1.8 mmol/L) for patients taking the maximally tolerated statin dose. If the LDL-C remains above the threshold level of ≥70 mg/dL or non-HDL cholesterol (defined as total cholesterol minus high-density lipoprotein cholesterol [a measure of total atherogenic lipoprotein burden in serum]) level ≥100 mg/dL (≥2.6 mmol/L), initiation of a PCSK9-I is reasonable if the cost/benefit ratio is favorable. Notably, the 2022 ECDP guidelines amended this recommendation and advocate for a target LDL-C <55 mg/dL and initiation of non-statin LLT if needed to achieve that goal for secondary prevention in this very high-risk population as well as those diagnosed with familial hypercholesterolemia (FH). Similarly, the European Society of Cardiology (ESC) 2021 guidelines advocate for a target LDL-C level of <55 mg/dL (<1.4 mmol/L) for those with very high-risk clinical ASCVD and recommend a target LDL-C level of <70 mg/dL (<1.8 mmol/L) for patients with only high-risk clinical ASCVD [30]. These guidelines also recommend a yet more ambitious LDL-C target of <40 mg/dL (1.0 mmol/L) for patients with ASCVD who experience a second vascular event within 2 years while taking maximally tolerated statin-based therapy.

It must be noted that the ESC guidelines, compared to those of the ACC/AHA which function within the age range of 40–75 years, base risk more on age group and utilize substantially lower risk stratification thresholds. Instead of the pooled cohort equations (PCE), the ESC guidelines estimate risk using the Systemic Coronary Risk Estimation 2 (SCORE2) and SCORE2-Older Persons (SCORE2-OP) risk algorithms. In addition to age, sex and traditional risk factors such as smoking status, systolic blood pressure and lipid measurements, common to both risk calculators, SCORE2 and SCORE2-OP factor in 4 distinct geographic regional risk categories (low, moderate, high, very high) and use age-, sex-, and region-specific risk factor values and ASCVD incidence rates. Important differences between the two sets of guidelines notwithstanding, the guiding principles for each are similar [31]. Large meta-analyses have shown that absolute reductions in LDL-C are directly proportional to reduction in ASCVD risk (i.e., “lower is better and lowest is best”) [17, 32]. This observation is consistent with the view that LDL particles constitute an important vascular toxin. According to the 2018 ACC/AHA guidelines criteria, the number needed to treat (NNT) with a moderate-intensity statin to prevent one ASCVD event in 10 years is 30, compared to 20 using high-intensity statin therapy [33]. Thus, focusing on initiation and titration of LLT is both cost-effective and clinically important to mitigate ASCVD morbidity and mortality.

## 3. Gaps in Care

Despite established guidelines, studies have shown significant gaps in care in patients with dyslipidemia (Table 1, Ref. [34, 35, 36, 37, 38, 39, 40, 41, 42, 43, 44, 45, 46]). Analysis of the Provider Assessment of Lipid Management (PALM) registry found that, among 5905 statin- eligible primary or secondary prevention patients from 130 cardiology and non-cardiology practices across the United States, up to one in four patients were not on a statin one year after the 2013 ACC guidelines were published. Moreover, even among those taking a statin, only 42.4% were on the recommended statin intensity [34]. Another study assessing a real-world primary prevention cohort of 282,298 patients at the University of Pittsburgh Medical Center, found that up to one in three statin-eligible patients based on the PCE were not prescribed a statin and, among those prescribed statins in the intermediate- and high-risk groups, the guideline-directed statin intensity (GDSI) was achieved in only 54% and 65.5% of patients, respectively, over the 6-year follow-up period [35]. Furthermore, a retrospective study using a commercially insured cohort of 134,008 patients without history of ASCVD, hospitalized for a first acute MI or stroke, found that <30% filled a prescription for a statin, ezetimibe, or PCSK9-I in the two years preceding their hospitalization [36]. This finding is consistent with an analysis of the 2015–2016 National Health and Nutrition Examination Survey data, which found that, among 32,278 patients, statins were prescribed to only 32.5% of patients with an estimated 10-year risk of ASCVD events ≥7.5% [37]. Suboptimal primary prevention may reflect inadequate patient identification and intervention using traditional ASCVD risk assessment tools [36].

**Table 1.** **Characteristics of a convenience sample of studies that demonstrate gaps in care in LLT for both primary and secondary prevention of ASCVD**.

Prevention type	First author, year of publication	Sample size of statin-eligible patients	Registry or Data Source	Prevention group inclusion criteria	Percentage of guideline-eligible patients on statin or other LLT (%)	Among patients taking a statin, percentage of patients taking GDSI (%)
Primary	Pokharel, 2016 [39]	911,444	Veteran Affairs	DM	68.3	85.5
Primary	Pokharel, 2016 [38]	215,193	PINNACLE	DM	61.6	Not reported
Primary	Virani, 2018 [40]	49,447	PINNACLE	LDL-C ≥190 mg/dL	58.5	54.5*
Primary	Saeed, 2021 [35]	282,298	University of Pittsburgh Medical Center	PCE-based 10-year ASCVD risk ≥7.5%	Intermediate-risk (7.5%–19.9%): 57	Intermediate-risk: 54
					High-risk (≥20%): 69	High-risk: 65.5
Primary	Sandhu, 2022 [36]	134,008	Optum de-identified Clinformatics DataMart	Prior to first acute myocardial infarction or stroke; no history of ASCVD	All patients: 29.5	Not reported
					DM: 45.0	
Both	Maddox, 2014 [41]	1,129,205	PINNACLE	Clinical ASCVD, LDL-C ≥190 mg/dL, DM, PCE-based 10-year ASCVD risk ≥7.5%	All: 67.6	Not reported
					ASCVD: 72.1	
					DM: 64.1	
					LDL-C ≥190 mg/dL: 70.7	
					ASCVD risk ≥7.5%: 64.5	
Both	Wong, 2016 [42]	1677	NHANES	Clinical ASCVD, LDL-C ≥190 mg/dL, DM, PCE-based 10-year ASCVD risk ≥7.5%	ASCVD: 63.7	Not reported
					DM: 43.2	
					LDL-C ≥190 mg/dL: 61.4	
					ASCVD risk ≥7.5%: 27.2	
Both	Navar, 2017 [34]	5905	PALM	Clinical ASCVD, LDL-C ≥190 mg/dL, DM, PCE-based 10-year ASCVD risk ≥7.5%	All patients: 74.7	All patients: 42.4
					ASCVD: 83.6	ASCVD: 47.3
					Primary prevention: 63.4	Primary prevention: 36.0
Both	Patel, 2019 [37]	32,278	NHANES	Clinical ASCVD, DM, PCE-based 10-year ASCVD risk ≥7.5%	DM: 60.2	Not reported
					ASCVD risk ≥7.5%: 32.5	
Secondary	Okerson, 2017 [43]	90,287	Optum Research Database	Clinical ASCVD	Pre-2013 Guidelines: 59	Pre-2013 Guidelines: 27
					Post-2013 Guidelines: 47	Post-2013 Guidelines: 31
Secondary	McBride, 2018 [44]	481,187	Veteran Affairs	CVD and/or PAD	All PAD: 79.0	All PAD: 40.9
					All CVD: 78.1	All CVD: 40.2
					PAD without CAD (with or without CVD): 69.1	PAD without CAD (with or without CVD): 28.9
					CVD without CAD (with or without PAD): 70.9	CVD without CAD (with or without PAD): 30.5
					PAD without CAD or CVD: 66.3	PAD without CAD or CVD: 26.4
					CVD without CAD or PAD: 69.9	CVD without CAD or PAD: 29.6
Secondary	Xian, 2019 [45]	3232	PALM	CVD and/or CAD	All: 84.3	All: 48.3
					CVD only: 76.2	CVD only: 34.6
					CAD only: 86.2	CAD only: 50.4
Secondary	Nelson, 2022 [46]	601,934	HealthCore Integrated	Clinical ASCVD	All: 50.1	All: 22.5
			Research Environment		CAD: 55.1	CAD: 49.8*
					CVD: 51.1	CVD: 43.2*
					PAD: 44.5	PAD: 37.5

Abbreviations: ASCVD, atherosclerotic cardiovascular disease; CAD, coronary artery disease; CVD, cerebrovascular disease; DM, diabetes mellitus; GDSI, guideline-directed statin intensity; LLT, lipid-lowering therapy; PAD, peripheral artery disease; PCE, pooled-cohort equation; NHANES, National Health and Nutrition Examination Surveys; PINNACLE, Practice Innovation and Clinical Excellence; PALM, Provider Assessment of Lipid Management; LDL-C, low-density lipoprotein cholesterol. *Number not explicitly stated in text; calculated as percentage of patients on high-intensity statin divided by percentage of patients on any statin.

Gaps extend beyond primary prevention. Within the American College of Cardiology’s National Cardiovascular Data Registry (NCDR) Practice Innovation and Clinical Excellence (PINNACLE) registry of participating cardiology practices, 38% of patients with DM [38] and 31.8% of patients with CAD [47] had no documentation of statin prescription, with significant practice-level variation. Furthermore, analysis of the Getting to an Improved Understanding of Low-Density Lipoprotein Cholesterol and Dyslipidemia Management (GOULD) study, a prospective, multicenter, observational registry of patients with clinical ASCVD, showed that only 17.1% of the 5006 enrolled patients had LLT intensification after 2 years, and two-thirds remained at an LDL-C level exceeding the 70 mg/dL threshold [48]. In addition, among patients with established ASCVD on statin therapy, over 50% discontinued the statin after only 6 months; moreover, longer-term adherence decreases progressively as a function of time [49]. Efforts to achieve target LDL-C levels for secondary prevention may be hindered by a combination of clinical inertia, low medication adherence and lack of access, among other factors [50].  Nevertheless, some progress in the use of statins for secondary prevention has been made, as evidenced by a retrospective cohort study that illustrated an increase in high-intensity statin therapy prescriptions after hospitalization for MI from 2011 to 2014 [51], illustrating the attainability of meaningful improvements in ASCVD prevention.

Differences in prescription rates of LLT based on race and sex are also well-documented [52]. For example, a study using the National Health and Nutrition Examination Surveys (NHANES) that included 3417 participants, representing 39.4 million US adults, found that overall statin use was significantly lower among Black and Hispanic as compared to White participants (20.0% vs 27.9%, *p* < 0.001, and 15.4% vs 27.9%; *p* < 0.001, respectively), as well as within each ASCVD risk strata [53]. Furthermore, a study examining the PALM registry found that, among 5693 statin-eligible participants, women were less likely than men to be prescribed any statin therapy (67.0% vs 78.4%; *p* < 0.001) or to receive the GDSI (36.7% vs 45.2%; *p* < 0.001) [54].

Significant heterogeneity in adherence to guideline recommendations has been demonstrated between clinics in the United States [39, 40, 55]. For example, in a study analyzing 911,444 patients with DM from 130 Veteran Affairs primary-care facilities, there was 20% facility-level variation in any statin therapy between 2 identical patients receiving care at 2 random facilities and 29% variation for moderate- to high-intensity statin use [39]. Furthermore, an analysis of 49,447 patients with LDL-C ≥190 mg/dL from the PINNACLE registry revealed significant practice-level variation in the proportion of patients receiving statin therapy, varying from just >10% of patients in some practices to >90% of patients in others [40]. Lastly, a retrospective cohort analysis using Medicare administrative claims and enrollment data found that, among 139,643 patients hospitalized for an acute MI, geographic region, rather than patient and hospital characteristics, was the most closely associated with high-intensity statin use after MI, leading to large treatment disparities [56]. It is evident that in both the primary and secondary prevention of ASCVD, there is vast underutilization of guideline-concordant statin use with inter-practice variability and healthcare inequities [57], representing a major gap in cardiovascular care and suggesting a need for national measures to promote uniform adherence to guidelines. Beyond the United States, a retrospective analysis of 2775 post-ACS patients in 7 European countries found that only 66% of the patients received a high-intensity statin therapy on discharge [58]. Moreover, among the 78% of patients with an LDL-C >70 mg/dL at the first follow-up visit, 41% had no change made to the LLT regimen. Considering the prevalence of these gaps in care, efforts by the World Heart Federation are underway to mitigate ASCVD burden globally [59], though the specific challenges are likely unique to each setting.

### Factors Contributing to Underutilization of Lipid-Lowering Therapies

Addressing this system-wide problem requires identification and exploration of potential root causes, which can be divided into patient-, clinician- and healthcare system-related factors [60]. Patient-related factors include medication non-adherence and intolerance to LLT. Given the systemic nature of ASCVD, patients taking LLT are commonly treated for several different cardiometabolic risk factors, such as hypertension, diabetes mellitus, heart failure or obesity, which can often lead to polypharmacy, a well-known cause of medication non-adherence [61]. Non-adherence may also be associated with a poor understanding of ASCVD risk and limited appreciation of the treatment benefits, which can be partly corrected for by enhanced clinician communication and data presentation. One study, including 3566 participants from the PALM registry, analyzed the effects of the clinician’s mode of data presentation on perceived risk and treatment willingness by randomizing participants to receive risk estimates using numbers only, a bar graph, or a face pictogram [62]. Respondents shown lifetime ASCVD risk were more likely to consider their risk “high to very high” than those presented with 10-year ASCVD risk or 10-year CVD death risk (70.1% vs 31.4% vs 25.7%, respectively; *p* < 0.0001). Treatment willingness was also highest for those shown their lifetime ASCVD risk (77.9% very willing) followed by those shown their 10-year ASCVD risk (68.1%) and their 10-year CVD death risk (63.1%; *p* < 0.0001), leading the authors to suggest that individuals are most affected by estimates that produce the highest absolute number. Additionally, the use of a pictogram for any given ASCVD risk led to lower risk perception and therapy willingness than a bar graph or no graphic. Similarly underscoring the importance of communication, another study analyzing a nationally representative sample of 6810 individuals with clinical ASCVD demonstrated that patients reporting poor patient-provider communication were at least 50% (OR 1.52; 95% CI, 1.26–1.83) more likely to report that they had not been prescribed or were not adherent to statin therapy [63].

Sensationalized media reports that occasionally inflate and dramatize side effects of statins have a deleterious impact on statin adherence. One study found that, among over 10 million patients in the United Kingdom already taking statins, patients were more likely to stop taking statins for both primary and secondary prevention after a period of widespread coverage of the debate over statin side effects across most major national media outlets (OR 1.11 [1.05 to 1.18; *p* < 0.001] and 1.12 [1.04 to 1.21; *p* = 0.003], respectively) [64]. Moreover, another study examining the effects of negative statin-related news stories on statin adherence and clinical outcomes among 674,900 Danish individuals found that the population attributable risk for early statin discontinuation was 1.3% for negative statin-related news stories [65]. Importantly, during follow-up, the multivariable adjusted HR for MI for individuals with early statin discontinuation was 1.26 (95% CI, 1.21–1.30) compared to individuals with continued use. Similarly, concerns over feared or perceived statin side effects were the most common reasons cited in the PALM registry for declining or discontinuing a statin, respectively [66].

Notably, in an analysis of 6579 (59.1%) of 11,124 patients who experienced a statin-related event leading to temporary statin discontinuation, over 90% were taking a statin 12 months after being rechallenged [67]. As others have critically pointed out, studies without a randomized blinded comparator group cannot distinguish between symptoms caused by chance versus those caused by a medication [68], highlighting the importance of improving healthcare literacy to better withstand periods of unregulated media reports. Additionally, the creation of a framework linking the academic community, or at least evidence-based consensus statements, with major search engines and social medial platforms to optimize the pursuit of high-quality, vetted healthcare information.

While maintaining freedoms of speech and press, has been proposed as a model to successfully reap the potential of highly accessible digital information while limiting the risk of misinformation dissemination [69].

Yet statin intolerance (SI), whether real or perceived, is a significant contributor to reduced long-term statin adherence. The National Lipid Association (NLA), recognizing the possibility of a “nocebo” effect (expectation of harm resulting in perceived side effects), requires that a minimum of two statins must be attempted, including at least one at the lowest approved daily dosage, for a diagnosis of SI to be made [70]. Though the incidence and prevalence vary by population, a meta-analysis including 176 studies with 4,143,517 total patients found that the overall prevalence of SI was 9.1% according to a range of diagnostic criteria (NLA, International Lipid Expert Panel, and European Atherosclerosis Society) [71]. Importantly, the Self-Assessment Method for Statin Side-effects or Nocebo [SAMSON] crossover trial, which randomized patients to receive atorvastatin 20 mg daily versus placebo and monitored daily symptom intensity for one year, found that 90% of the symptom burden elicited by a statin challenge was also elicited by placebo (i.e., simply taking a pill correlated with development of muscle symptoms) [72]. Thus, the importance of not interpreting symptoms as indicative of pharmacologic causation cannot be overstated.

For patients with SI, alternatives to statins show promise. For example, the Goal Achievement After Utilizing an Anti-PCSK9 Antibody in Statin Intolerant Subjects 3 (GAUSS-3) RCT, which randomized 218 patients with SI and an entry mean LDL-C level of 219.9 mg/dL to receive either ezetimibe or evolocumab, found that while both agents were effective at lowering LDL-C, evolocumab was significantly superior (absolute reduction: 102.9 mg/dL vs 31.2 mg/dL; *p* < 0.001; mean percent reduction: 52.8% [95% CI, 55.8–49.8] vs 16.7% [95% CI, 20.8–12.5]) [73]. Furthermore, the recently published Cholesterol Lowering via Bempedoic Acid [ECT1002], an ACL-Inhibiting Regimen (CLEAR) Outcomes trial, a RCT that enrolled 13,970 patients with SI, demonstrated that patients who received bempedoic acid had a significantly lower incidence of a composite primary end-point of major adverse cardiovascular events compared to placebo (HR 0.87 [0.79–0.96]; *p* = 0.004) [74]. Furthermore, the emergence of a range of novel therapies to add to the LLT armamentarium supports the notion that, even in patients who are statin intolerant, a LLT alternative will often be available [75].

Regarding clinician-related factors, Nanna *et al*. [66] analyzed the PALM registry and noted that, relative to practices with the lowest or mid-tertile use of statins, practices in the highest tertile were characterized by a significantly greater number of providers (11 vs 4 vs 2; *p* < 0.001), were cardiology-based as opposed to primary care-based (68.0% vs 48.0% vs 12.5%; *p* < 0.001), and had physicians (in contrast to advanced practice providers) constituting >90% of the practice compared to less than three-quarters of the providers in the lowest tertile practices. In addition to infrastructure, clinicians in the highest tertile practices more frequently reported adopting the latest ACC/AHA Cholesterol Guidelines (80.2%) compared with mid- (67.8%) or lowest tertile practices (59.3%) (*p* = 0.003) and were more likely to agree or strongly agree with the statements that statins are safe (72.8% vs 69.8% vs 56.6%, *p* < 0.05) and prolong life (79.0%, vs 72.1% vs 53.7%; *p* < 0.001). Likewise, another study evaluated physician knowledge of updated guidelines by asking 67 specialist physicians to analyze anonymized records on up to 50 patients with diabetes and dyslipidemia and specify perceived cardiovascular risk, LDL-C targets, and the suggested refinement in LLT [76]. Physician-based assessments of cardiovascular risk and of LDL-C targets were misclassified in 34.7% of the records as compared to guideline recommendations. Furthermore, the United States Preventive Services Task Force’s conclusion that there is insufficient evidence to recommend initiation of statin therapy for ASCVD primary prevention among adults aged 76 years and older [77], mostly owing to a lack of dedicated RCTs including this demographic, may be related to underuse of effective interventions among healthy older adults [78]. Lastly, lack of clinician knowledge of important statin drug interactions may possibly lead to adverse effects, which might play a role in statin discontinuation [79].

It is worth emphasizing that inadequate physician knowledge regarding LLT not only limits appropriate LLT prescriptions but may also generate confusion among patients due to inconsistencies between healthcare providers. In pursuit of effective clinician education, numerous efforts are underway to improve both the passive diffusion of guidelines, with implementation of modular knowledge chunk format and lower word limits, as well as active dissemination of guidelines, which include derivation of guidelines, audit and feedback, academic detailing, decision mapping, mass media support, and financial incentives [80].

As would be expected, the aforementioned treatment gaps have both medical and financial costs. In a propensity-matched retrospective observational study comparing 5,190 patients with SI to 15,570 patients taking statins, patients in the non-statin group experienced a higher risk for revascularization procedures overall (HR, 1.66; 95% CI, 1.36–2.02; *p* < 0.0001) and incurred higher healthcare costs (cost ratio, 1.20; 95% CI, 1.11–1.28; *p* < 0.0001) [81]. Furthermore, in a study assessing the association between LDL-C and longer-term cardiovascular events after percutaneous coronary interventions among 47,884 patients, those who achieved an LDL-C level <70 mg/dL at 6 months had a cardiovascular event rate of 55.2/1000 person-years, compared to 60.3/1000 person-years for the 70 to <100 mg/dL group and 94.0/1000 person-years for the ≥100 mg/dL group [82]. A similar study investigated the association between LDL-C changes and prognosis after an MI. Among 40,607 patients followed for a median of 3.78 years, patients with larger LDL-C reductions (1.85 mmol/L, 75th percentile) compared with smaller reductions (0.36 mmol/L, 25th percentile) had lower HRs for all outcomes including all-cause mortality (0.71 [0.63–0.80]), cardiovascular mortality (0.68 [0.57–0.81]), MI (0.81 [0.73–0.91]), ischemic stroke (0.76 [0.62–0.93]), heart failure hospitalization (0.73 [0.63–0.85]), and coronary artery revascularization (0.86 [0.79–0.94]) [83].

From a healthcare systems point-of-view, access to care, including clinic visits, medications, costs and pharmacy availability, has been shown to correlate with LLT adherence [84, 85, 86]. The importance of financial barriers to medication adherence is evidenced by the National Health Interview Survey (2013–2017), which found that, in 14,279 individuals with clinical ASCVD, one in eight attributed medication non-adherence to cost [84]. Apart from general healthcare costs, newer LLT such as PCSK9-I pose a particular challenge regarding insurance approval, which certainly translates to clinical outcomes. In a review of 139,036 patients who were prescribed PCSK9-I, 61% of patients had their initial PCSK9-I prescription claims rejected, and this group was found to have a higher adjusted HR for a composite cardiovascular event outcome compared to patients with their initial PCSK9-I prescription claims approved (HR 1.10; 95% CI, 1.02–1.18; *p* = 0.02) [87]. In this vein, many argue that there are clear unintended consequences of the need for prior authorizations for PCSK9-I, including heavy administrative burden and indiscriminately high rejection rates, and advocate for a redesign of the prior authorization process [88]. In addition to medication access, access to care—the ability to participate in regular follow-up—has also been demonstrated to correlate with both statin prescriptions and adherence [89, 90]. Irrespective of access to medications and care, however, disparities in statin prescription and use based on patient factors such as race/ethnicity, sex, age, socioeconomic status, and comorbidities have been consistently reported [91, 92]. Furthermore, analysis of the PINNACLE registry demonstrated that patients in the wealthiest quintile had a small but significantly higher likelihood of appropriate statin therapy compared to patients in the poorest quintile (OR 1.03; 95% CI, 1.01–1.04) [93]. These findings highlight the need for awareness of all forms of implicit and explicit bias to ensure equitable care in addition to addressing flaws inherent in the healthcare system.

## 4. Lifestyle Therapies

The ACC/AHA guidelines emphasize that, whether as a precursor or adjunct to pharmacologic therapies, lifestyle interventions—specifically, diet, weight control and physical exercise—are at the forefront of ASCVD risk reduction [20]. Likewise, avoidance of tobacco smoke [94] and ensuring optimal sleep duration [95] are both critical for cardiovascular health. Considering strong data showing that ASCVD risk can be reduced by diet [96], both the ACC/AHA and ESC guidelines recently gave a class I recommendation for the consumption of a plant predominant diet [30, 97]. Similarly, the American Society for Preventive Cardiology defines a healthful diet as one with a predominance of fruits, vegetables, legumes, nuts, seeds, plant protein and fatty fish, and a paucity of saturated fat, dietary cholesterol, salt, refined grains, and ultra-processed food [98]. Recognizing the acute care setting as an opportunity to improve patient nutrition and lifestyle, hospitals are beginning to implement initiatives to increase awareness of optimal dietary patterns during inpatient admissions and promote “teachable moments” to guide patients toward adopting more healthful lifestyles [99]. Furthermore, leveraging electronic health records (EHRs) to make the “healthy choice” the easy choice during a hospital admission, may facilitate positive lifestyle change. For example, an admission order template can make a healthful diet order the default, with associated education for the patient and reinforcement from other providers. Considering that about 1 in 7 US adults with ASCVD experience food insecurity [100], some advocate for political change via a rerouting of government subsidies towards fruit and vegetable programs to incentivize production and promote affordable consumption [101]. These are just some of the many ways in which lifestyle therapies are currently being pursued to mitigate ASCVD burden.

## 5. Cholesterol Measurement as a Quality Metric  

Value-based care is an accepted pillar of healthcare. Since its development in the 1960s, quality improvement and quality measures have been central to ensuring health care facilities provide quality care to patients [102]. To encourage quality healthcare delivery in all levels of healthcare, governmental and non-profit agencies, such as the Center for Medicare-Medicaid Services (CMS) and National Center for Quality Assurance (NCQA), publish guidelines that define quality metrics for the healthcare system.

Lipid measurement and treatment were established as a quality measure by the NCQA for reimbursement in 2001. These have historically been modeled after the National Cholesterol Education Program and its Adult Treatment Panel (NCEP-ATP) [103]. The 2001 NCEP-ATP III guidelines established LDL-C as a treatment goal per level of risk, initially using the Framingham risk score to identify low-, moderate- and high-risk categories for patients. This was the first national example of health care organizations collecting data and developing strategies to ensure primary prevention for ASCVD [104].

A decade ago, however, measurement of LDL-C levels was removed as a quality metric from guidelines. This change ensued after the publication of the 2013 ACC/AHA Cholesterol treatment guidelines, which recommended management by using statin therapy at various intensities based on risk level, without a target LDL-C level. Despite removal of a target LDL-C, however, these guidelines still recommended measurement of LDL-C as a Class I recommendation to monitor response and adherence to LLT. Misinterpretation of this guideline led to the removal of LDL-C target level and LDL-C monitoring for patients on LLT across multiple NCQA and CMS guidelines including DM, FH and ASCVD risk [103].

**Fig. 1.**
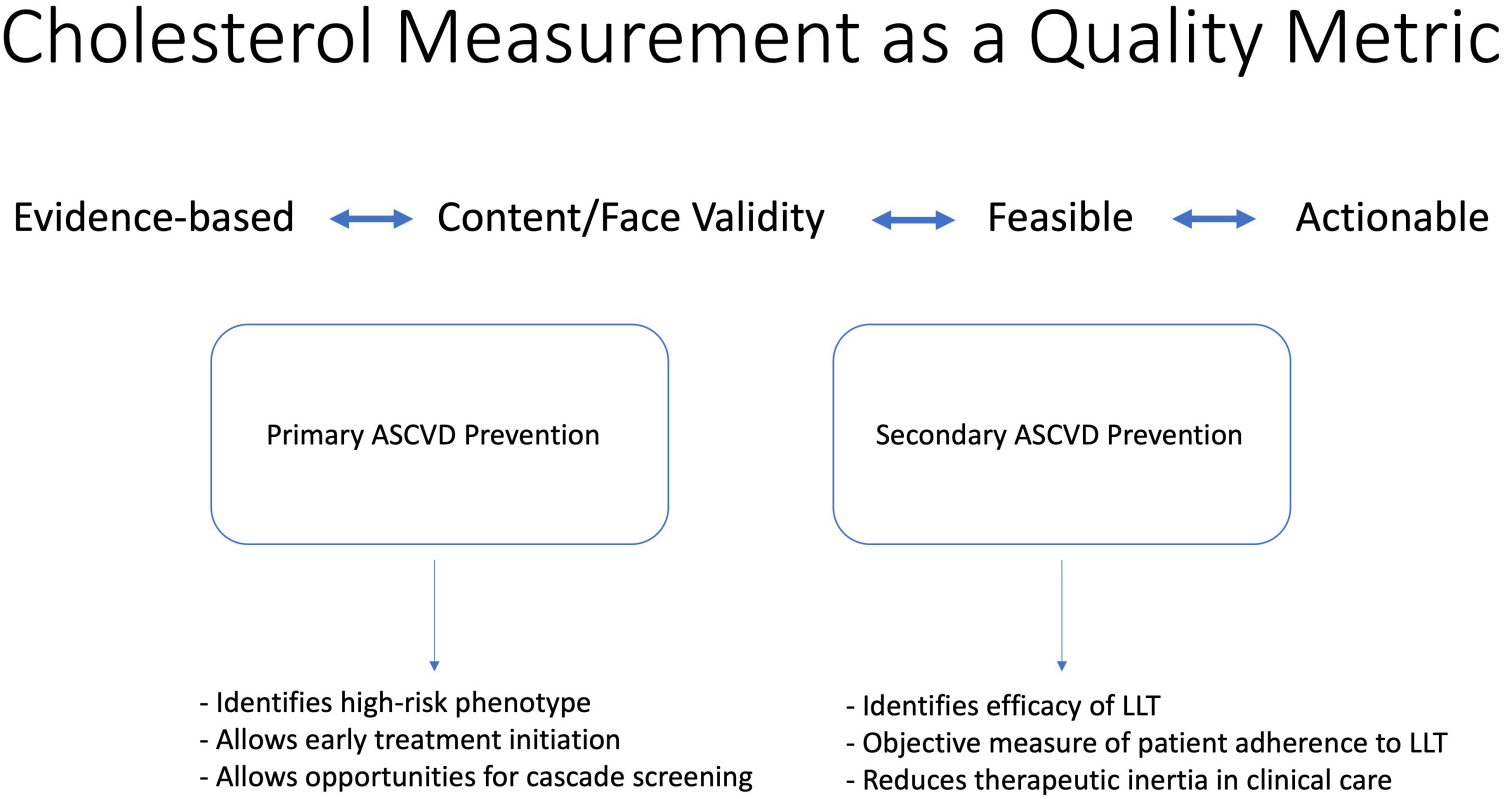
**Value of cholesterol as a quality metric**. Abbreviations: ASCVD, atherosclerotic cardiovascular disease; LLT, lipid-lowering therapy.

New data have emerged that support the re-establishment of monitoring LDL-C levels after initiating or modifying treatment (Fig. 1). For example, in the JUPITER trial (Justification for the Use of Statins in Prevention: an Intervention Trial Evaluating Rosuvastatin [JUPITER] trial), efficacy of statins was studied in patients who were on a fixed dose of rosuvastatin 20 mg daily. There was a significant heterogeneity in LDL-C response to statins, with some patients achieving no reduction or even an increase in levels [105]. While this discrepancy may, in part, be due to differences in lipid metabolism and drug pharmacokinetics, medication nonadherence is also likely contributory. As abovementioned, reasons for statin nonadherence are multifactorial [106]; nevertheless, observational studies have found that routine LDL-C monitoring is associated with increased adherence [107]. For example, one retrospective cohort study found that in a group of 19,422 patients, those scheduled for follow up visits with LDL-C monitoring were 45% more likely to be adherent than patients without scheduled follow up visits [108]. In part for these reasons, the 2018 AHA/ACC/Multisociety cholesterol treatment guideline (similar to the 2013 ACC/AHA Cholesterol guideline) currently recommends monitoring LDL-C levels 4–12 weeks after initiation or dose adjustment to assess statin efficacy and help guide the decision of whether newer non-statin therapies should be added as a class 1A indication, with follow up every 3 to 12 months thereafter. Despite evidence-based guidelines maintaining the importance of measuring LDL-C levels to assess efficacy, adherence and the need for additional LLT, quality metric publications have not yet reinstated LDL-C monitoring as a quality measure.

In addition to conventional lipids, elevated apolipoprotein B (apoB)-containing lipoproteins, including lipoprotein(a) [Lp(a)], are also known to have a causal relationship with ASCVD risk, even in the setting of normal or low LDL-C [109]. As such, the ESC guidelines recommend testing for Lp(a) at least once in each adult’s lifetime [110] while the ACC/AHA guidelines consider family history of premature ASCVD a relative indication for testing [20]. Despite these recommendations, testing remains remarkably uncommon. A retrospective analysis of >5.5 million adult patients across 6 academic health systems in California found an overall testing prevalence of 0.3%, <4% among patients with CVD and 3.3% among patients with a family history of ASCVD [111]. In addition to reinstating LDL-C monitoring as a quality metric, testing for Lp(a) must also be emphasized as an important component of ASCVD risk stratification.

In a similar vein as reinstating LDL-C monitoring as a national metric, it seems reasonable to advocate for lipid testing to be included as part of the expert consensus or conventional practice of precatheterization care [112]. In the setting of significantly inadequate LLT utilization and optimization, diagnostic angiograms represent a unique opportunity to pair metabolic findings with clearly observable plaque burden. Instead of dissociating catheterization findings from lipid levels, relegating the latter for outpatient follow-up at a future time, presenting the two elements as fundamentally two sides of the same coin can engage patients to and encourage them to take a more active role in their health care.

## 6. Advances in Screening

Since the development of the Framingham Risk Score, researchers continue to develop better ASCVD predictive models [113]; however, even with the incorporation of different baseline characteristics, multiple studies have shown how each of these risk scores under- or over-estimates risks for certain populations. The 2018 ACC/AHA guidelines on Use of Risk Assessment Tools to Guide Decision-Making in the Primary Prevention of Atherosclerotic Cardiovascular Disease allow for risk modifiers and inclusion of coronary artery calcium (CAC) testing to better understand risk for people in the low and intermediate risk categories [113].

The 2018 guidelines also expanded screening for FH, an inherited disease that impacts approximately one out of every 250 people, though a query of the Family Heart Database found that an ICD-10 code (International Classification of Diseases, Tenth Revision [ICD-10]) for FH was found for only 26% of the 277 included individuals with severe hypercholesterolemia [114, 115]. These patients have an increased risk of ASCVD compared with patients without FH [116]; however, screening patients for FH is not included in Health Effectiveness Data and Information Set (HEDIS) measures for reimbursement [103]. There are multiple scoring systems that have been developed to diagnose FH; however, no universal consensus statement exists. The AHA Criteria that developed FH diagnostic categories is a more simplified approach to making the diagnosis and is easier to implement in clinical practice [117, 118]. As discussed in Section 10, machine learning with the FIND FH (Flag, Identify, Network and Deliver FH) program has been the newest strategy to identify these high-risk patients.

## 7. Imaging of Coronary Atherosclerosis to Optimize LLT Utilization 

CAC scoring was developed by Agatston and Janowitz in the 1980s using gated non-contrast electron beam computed tomography (EBCT) to identify calcium with attenuation greater than a 130 Hounsfield unit threshold, with an area of at least 1 ${\mathrm{mm}}^{2}$. Other scores have been developed, including calcium volume score, calcium mass score and calcium density score [119]. Moreover, CAC can be calculated from non-gated computed tomography (CT) using an easily performed ordinal calcium score or the Agatston score [120]; thus, the Society of Cardiovascular Computed Tomography has recommended to report CAC on all non-gated chest CTs [121]. Unfortunately, this recommendation is not yet the standard of care. One reason may be because CAC scoring calculation is time-consuming; however, this is likely to change with the benefits of emerging artificial intelligence (AI) and deep learning technologies. Notably, a CAC score of 0 has been found to be one of the strongest negative risk factors for ASCVD, known as the “power of zero” [122], allowing for de-escalation of risk. One analysis of the MESA study downgraded risk levels for 44% of patients eligible for statins based on CAC = 0, with 4.2 ASCVD events per 1000 person years [123]. Moreover, it can identify individuals without prior ASCVD at an equivalent risk of major cardiovascular events to those with established ASCVD [124]. Importantly, CAC performs best when used in conjunction with risk estimators [123, 125].

Coronary CT angiography (CCTA) allows identification of specific coronary atherosclerosis phenotypes and has been used to identify and risk stratify both asymptomatic and symptomatic patients (though use in asymptomatic patients is currently only within the research realm). CCTAs are the recommended test for risk stratification for symptomatic patients with low-to-intermediate risk (15–50%) and can provide quantitative and qualitative data about the type of plaques patients may have [126]. Regarding symptomatic patients, the Scottish Computed Tomography of the Heart (SCOT-HEART) trial found that in a cohort of 4146 patients with stable chest pain, patients that underwent CCTA demonstrated a significantly lower death rate without a significantly higher rate of coronary angiography or revascularization (2.3% vs 3.9% in standard of care; 95% CI, 0.41–0.84; *p* = 0.004) [127]. The patients randomized to the CCTA group were also more likely to have preventive therapies started (OR 1.4; 95% CI, 1.19–1.65). Beyond symptomatic patients, we now have 3 large-scale population-based studies on CCTA imaging in asymptomatic individuals (Swedish CArdioPulmonary bioImage Study [SCAPIS] [N = 25,182] [128], Miami Heart [N = 2459] [129], and Copenhagen General Population Study [N = 9533] [130]). The SCAPIS trial, which analyzed 25,182 asymptomatic patients without known CAD who underwent CCTA, found atherosclerosis in 42% and >50% stenosis in 5.2%, illustrating that subclinical atherosclerosis is common in the general population [128].

## 8. Televisits 

In the last few decades, the use of digital technologies for health purposes has drastically increased, illustrating their potential for improving the quality of care for patients, reducing hospital readmissions and saving costs for providers and patients [131, 132]. Telemedicine is defined as the use of information and communication technologies to deliver medical care and health service from a distance [131, 133]. In the United States, the earliest application of telemedicine was performed by the National Aeronautics and Space Association in 1960, using medical monitors to observe the health of astronauts in flight [131]. This laid the foundation for new research using telemedicine which mainly addressed shortages of specialty care in rural areas [131, 133, 134].  In the last 20 years, the use of telehealth for ASCVD prevention has grown tremendously. Some programs use nurse-led interventions to improve LLT adherence or educate patients regarding lifestyle modifications [135]. Furthermore, home-based cardiac rehabilitation programs were implemented using heart rate telemonitoring and telecoaching to improve adherence to exercise, dietary modifications, medical treatment, and to positive lifestyle changes [136, 137].

The COVID-19 pandemic allowed for the development and maturation of several digital technologies that can be applied to tackle major clinical problems and diseases [138, 139]. Regarding dyslipidemia, the use of telemedicine for lipid management is developing, though research on this topic has not shown clear outcomes. For example, one systematic review found that the use of telehealth had a positive to neutral impact on improving a composite outcome of lipid metrics, medication adherence to LLT, or lipid management education [133]. Televisits increase the amount of patient data collected, supplying clinicians with a more complete understanding of each individual patient, as well as supplying the provider with a better understanding of the patient’s home environment. It also permits faster therapeutic titrations and prescriptions according to the updated metrics [133].  The burden of large amounts of data will require AI-driven solutions to optimize data management and utilization. Without assistance of data filtering, physicians could find themselves overwhelmed by information.

In a prospective cohort study, 375 patients with diabetes were randomized to telehealth consultation in addition to standard antidiabetic therapy versus usual care to reduce LDL-C levels [139].  The standard treatment group had considerably higher levels of plasma LDL-C than the telehealth consultation group after just 1 month (110 vs 93.1 mg/dL, *p* < 0.001). The authors concluded that telehealth consultation may be a suitable complement to pharmacologic therapy for diabetic patients to assist in the management of proatherogenic dyslipidemia and postprandial glucose variability. Similarly, one hospital system in Spain used televisits during the COVID-19 pandemic to rapidly uptitrate LLT for patients following ACS admissions [140]. Patients were prescribed 80 mg atorvastatin on hospital discharge with a scheduled lipid panel one month thereafter. Following those one-month results, televisit appointments were used to discuss the results and advance the LLT regimen if patients did not reach the target goal of <55 mg/dL. This process of a subsequent lipid panel followed by telehealth visit one month later was repeated for further medication adjustments if indicated. In this group of 346 patients, the mean LDL-C dropped 55% from admission rates, with 95% of patients achieving LDL-C below 70 and 82% achieving LDL-C below 55 mg/dL in an average of 3.2 months.

Other studies, in contrast, did not find significant improvements in outcomes. For example, the use of telehealth counseling for risk factor management and lifestyle modifications in individuals at high-risk for cardiovascular events compared to brief preventive counseling did not show significant between-group differences for reduction of cholesterol levels and 10-year ASCVD risk score [141]. Nevertheless, telehealth counseling for 6 months did improve adherence to exercise and dietary changes. As more data accrues on which forms of telemedicine yield the greatest improvement in clinical outcomes, optimization and implementation of the most evidence-based programs has the potential to significantly improve the delivery of preventive measures with potential to significantly decrease ASCVD burden.

## 9. Digital Technologies

Online platforms and mobile applications can enhance the way physicians and other allied healthcare workers manage patient care. For example, Virani *et al*. [142] showed in a multi-centered RCT how electronic alert reminders sent to physicians can improve statin initiation and titration in appropriate patients. The alert reminders included type of ASCVD diagnosis, statin dose, date of last refill, statin associated side-effects, and management guidelines. Furthermore, the Corrie Health Digital Platform, an application developed using the Health Belief Theory and social cognitive theory, allows patients with recent MIs to start understanding and managing their diagnosis while still hospitalized and in the post-acute care transition at home. The platform, which integrates a smartphone app with a smartwatch and blood pressure monitor to provide patient tracking of medications, vital signs, education and care coordination, decreased 30-day hospitalizations post-MI by 52% compared with the control group [143]. Another smartphone application that automates calculation of LDL-C by utilizing the Martin-Hopkins equation can calculate LDL-C levels more accurately than the previous Friedewald equation [144, 145].

A large barrier to mobile health applications is patients’ lack of access to mobile phones. This was addressed by the iCorrie Share study, which provided participants with a loaner iPhone; at the end of the study, 72% of the phones were returned following a successful expansion of access to an impactful intervention to a diverse patient population [146]. Several other RCTs assessed other forms of digital technologies with promising results. For example, motivational text messages helped patients increase physical activity in the mActive trial [147] and showed slight improvement in LDL-C levels in the Tobacco, Exercise and Diet Messages (TEXT ME) trial [148]. The benefits of using online platforms and mobile applications in patient care are supported by a 2021 systematic review and meta-analysis [149], though many of the applications included were designed for the trials and are not yet commercially available. Considering that there are many areas in the United States that lack adequate broadband internet access and/or cell towers, an obvious rate-limiting step for digital technologies, efforts to expand access are essential to enable all of society to reap the benefits of technological progress and prevent a digital divide.

## 10. Artificial Intelligence

Artificial intelligence and machine learning can be used as another strategy to address gaps in care by combining information from EHRs, cardiovascular imaging, wearable sensors and social determinants of health to provide enhanced risk evaluations for individuals [150]. Myers *et al*. [151] developed a machine learning program called FIND FH that was able to detect 87% in a national database and 77% in a health care delivery system dataset as having high enough suspicion for FH to trigger screening and treatment. Similarly, Eng *et al*. [152] developed a machine learning program to generate CAC scores from both gated and non-gated CTs. This method of opportunistic screening is an effective way to obtain critical data regarding ASCVD risk and comes at no additional cost (other than the software) or radiation penalty. This machine learning-driven CAC scoring was near perfect when compared with board-certified diagnostic radiologists’ readings (mean difference in scores = –2.86; Cohen’s Kappa = 0.89; *p* < 0.0001) and was done in a significantly shorter amount of time (3.5 seconds vs 261 seconds for manual) [152]. As discussed above, Sandhu *et al*. [120] used elevated CAC scores identified by machine learning on non-gated chest CTs and randomized groups to either have a notification sent to the primary care clinician and patient or proceed with usual care. Prescriptions for statins were significantly greater for patients in the notification arm compared to usual care (51.2 vs 6.9%; *p <*0.001) [120]. Even though, as abovementioned, all non-gated chest CT’s reports should ideally include an evaluation of CAC, this is still not the standard in the real-world. Automated scores and generation of referral lists for those with significant CAC could potentially lead to improvements in the identification of patients at risk. As a caveat, patient notifications should be deployed with caution as notifications without the appropriate access to care in response can generate significant anxiety.

Causal AI has also recently been used to quantify individual lifetime risk for cardiovascular disease and provide recommendations regarding the degree to which LDL-C and systolic blood pressure should be reduced to effectively decrease ASCVD risk. Ference *et al*. [153] built an AI model that incorporated LDL-C and systolic blood pressure in discrete time units of exposure to evaluate how lifetime risk impacted outcomes.  These authors showed that even patients with a very high genetic predisposition to heart disease can overcome that genetic predisposition by optimizing blood pressure and LDL-C levels. The rapid expansion of AI to all aspects of medicine is also not without risks, as it is known that AI can harbor biases that further expand the existing disparities in healthcare for historically underserved populations. Bearing in mind that AI can “compound existing inequities in socioeconomic status, race, ethnic background, religion, gender, disability or sexual orientation to amplify them and adversely impact inequities in health systems [154]”, developers and regulators of AI must adhere to the strict safety regulations already established for research in the medical field [155].

## 11. Multifaceted Approach 

A multifaceted approach is needed to manage and care for patients at risk and with established ASCVD in which multiple risk factors need to be addressed and multiple barriers overcome to improve the management of dyslipidemias and decrease ASCVD risk.  Patients, health professionals, and institutions have respective roles and responsibilities in achieving health goals. One example of this is the Cardiac Collaborative Care Service (CCCS), a multi-disciplinary program developed by Kaiser Permanente of Colorado consisting of a nursing team and a pharmacy team. The team works with patients, primary care physicians, cardiologists, and other health care professionals to coordinate cardiac risk reduction strategies for patients with ASCVD, including lifestyle modification, medication initiation and adjustment, patient education, laboratory monitoring, and management of adverse events. In a retrospective, observational cohort of 8014 patients, screening for cholesterol increased from 66.9% to 97.3% at the end of the evaluation period. After a mean follow-up duration of 2.3 years, the number of patients attaining the predefined LDL-C goal of <100 mg/dL increased from 25.5% to 72.7%, of whom almost 85% were only receiving statin monotherapy [156]. The average LDL-C for those patients decreased from 119 mg/dL to 89 mg/dL. Moreover, in an analysis of patients enrolled from 1996 to 2004, implementation of CCCS for secondary ASCVD prevention was associated with a reduction in all-cause and ASCVD-related mortality as well as reduced health care expenditures [157].

A more recent study from the Kaiser Permanente of Colorado employing a similar program for home-based cardiac rehabilitation revealed significant fewer hospitalizations at 12 months among participants [158]. The benefits observed from the CCCS studies support widespread emulation and implementation. Similarly, a multifaceted approach, coordinated between non-licensed navigators, pharmacists, and cardiovascular clinicians, was implemented at the Mass General Brigham system to control hypertension, LDL-C levels, or both in a cohort of 10,830 patients. After program enrollment, measurements of blood pressure and LDL-C were taken at 6 and 12 months. Patients in the remote medication management experienced a reduction in LDL-C by a mean (SD) 35.4 (43.1) and 37.5 (43.9) mg/dL at 6 and 12 months, respectively, compared to those in the education-only cohort who experienced a mean (SD) reduction in LDL-C of 9.3 (34.3) and 10.2 (35.5) mg/dL at 6 and 12 months, respectively (*p* < 0.001) [159].

## 12. Conclusions

**Fig. 2.**
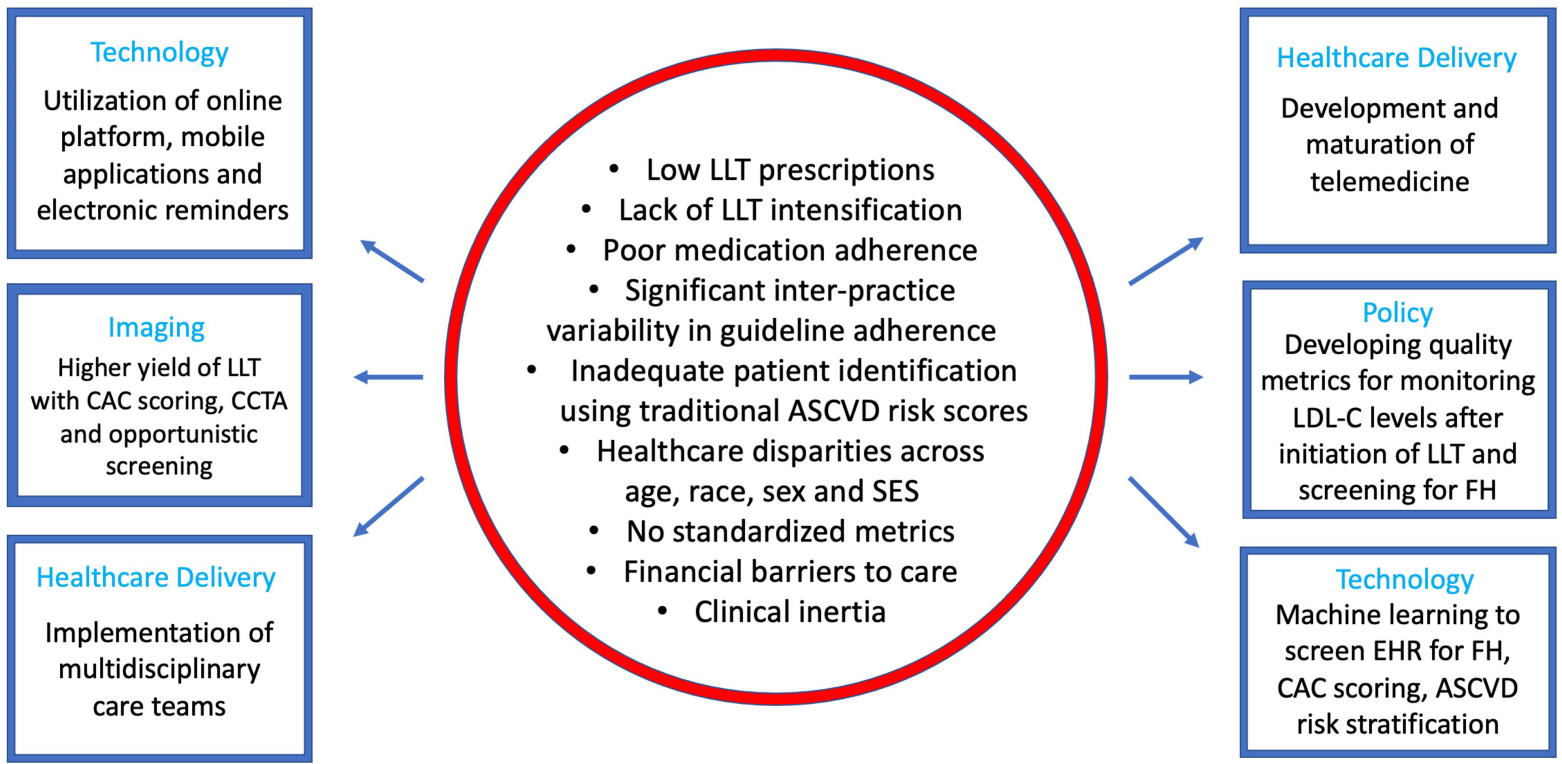
**Graphical depiction of treatment gaps and opportunities in lipid management**. ASCVD, atherosclerotic cardiovascular disease; CAC, coronary artery calcium; CCTA, coronary computed tomography angiography; EHR, electronic health record; FH, familial hypercholesterolemia; LDL-C, low-density lipoprotein cholesterol; LLT, lipid-lowering therapy; SES, socioeconomic status.

The confluence of programmable EHRs, multidisciplinary care teams, new digital technologies and a surge in telemedicine has the potential to dramatically improve the management of dyslipidemia, and thus reduce ASCVD burden, on a population scale [160] (Fig. 2). We believe that a crucial first step in reducing ASCVD burden is establishing national quality metrics that are aligned with current clinical recommendations. The imperative to reinstate LDL-C measurement as a performance measure for ASCVD patients in managed care organizations represents a hurdle that must be overcome to effect meaningful change. This needs to be incorporated into the Universal Foundation - a quality measure Jacobs *et al*. [161] recently urged the various CMS quality affiliated programs to adopt. Likewise, given the large-scale impact of national quality measures, including FH screening in the HEDIS measures, with a recommendation to initiate high-intensity statin therapy for those with LDL-C >190 mg/dL, is critical to address this high-risk population. Since LDL-C directly correlates with ASCVD risk and statins are the first-line class of LLT, any measure that will increase appropriate statin prescription, intensification and adherence should be contemplated. The well-documented potential of rechecking lipid profiles to reduce therapeutic inertia, increase evidence-based statin prescribing, and increase statin adherence compellingly support the notion to re-establish LDL-C levels as a metric of quality care.

From this national metric, each health system can then use this standard of care to develop best screening and implementation practices that are modeled to address the barriers and fit the needs of the community they serve. The establishment of a lipid champion or specific lipid or cardiometabolic clinic in each health system could serve as a center of excellence to be emulated [162]. The European Atherosclerosis Society has done this with the initiation of the Lipid Clinics Network, and there are independent certified lipid specialists who can be found on the NLA or Family Heart Foundation websites. This network provides not only an infrastructure for online educational activities and training but also for local webinars and global surveys as a unique way to identify and address gaps in knowledge and needs. For example, a recent international survey among participants in the Lipid Clinics Network revealed the extent to which measurement of Lp(a) remains an underused practice and explored possible underlying reasons [163]. This effort identified three key underlying factors; namely, lack of reimbursement, lack of standardization of testing and lack of therapeutic agents specifically targeting Lp(a). This exchange of real-life experiences, particularly between a designated group of experts in the field, has significant potential to raise awareness of important practical issues and thereby promote changes in healthcare policy. This is a relatively new development in 2021 and no studies have been done to evaluate the network’s effectiveness [164]; however, in addition to invaluable dialogue between experts, patients are more likely to have PCSK9-I prescribed and approved when evaluated by cardiologists or lipidologists [66], as discussed above, which will likely correlate with clinical outcomes.

Another proposed solution to increase the use of statin therapy in eligible patients, which may be particularly of use in regions with less access to care, is to reclassify statins as nonprescription over-the-counter drugs [165]. With the aid of an at-home Web-based application to assess appropriateness for treatment with rosuvastatin 5 mg, participant self-selection was found to largely agree with clinician selection [166], supporting the notion that broader access to statins could have a significantly positive public health impact, at least as an initial step prior to patients accessing more comprehensive care.

The establishment of best practices for primary prevention that utilizes EHRs to identify suitable patients to be engaged in multiple strategies to ensure medication adherence is achieved is essential for primary prevention [167]. Secondary prevention should start as soon as the patient is admitted to the hospital, ensuring adequate access to LLTs before discharge with close follow-up thereafter. At some hospitals, new initiatives of “meds to bed” programs for PCSK9-I have started to secure the bedside delivery before discharge for the very-high risk patients, when appropriate, while newer data suggests that upfront combination LLT can improve long-term outcomes for patients with ASCVD. Additionally, institutional protocols for precatheterization lipid assessments can catalyze enhanced patient engagement in their own care, with potential to improve medication adherence, lifestyle modifications, or both. Furthermore, every available opportunity to promote positive lifestyle changes for both primary and secondary ASCVD prevention must be seized. We believe that combining the above strategies, leveraging and integrating digital solutions within evolving systems of care, can effectively mitigate ASCVD by increasing guideline-directed prescription and adherence to LLT.

Though this review highlights a great number of opportunities to optimize lipid management in the 21st century, their practical implementation undoubtedly depends upon both the patient population being served and the resources available. In addition to, collectively as a community of clinicians, advocating for the re-establishment of LDL-C monitoring as an international quality metric, each practice must determine which interventions are most likely to be effectively carried out in their unique healthcare landscape and within their budget. For practices with greater financial constraints, focusing on the evolution of healthcare delivery would be prudent. For example, the formation of multidisciplinary care teams is simply a matter of reorganizing and integrating pre-existing providers from various specialties to promote more holistic and patient-centered care. Likewise, for regions with widely accessible broadband internet access, utilization of telemedicine as a complementary therapeutic modality for patients already being treated pharmacologically for dyslipidemia can be a relatively low-cost way of improving outcomes. Increasing the use of mobile applications and electronic reminders, too, likely do not carry too onerous a cost, though third-party subscription fees may vary depending on the services or technologies being offered. Given the recent data showing the benefits of CCTA imaging, centers with greater financial means should prioritize ensuring there is an adequate quantity of CT machines to match the growing number of patients who will be referred for this imaging modality. Lastly, the potential to improve long-term morbidity and mortality through advanced technologies and machine learning by extracting valuable insights from previous imaging and EHRs may likely outweigh the higher upfront costs. Regardless of which of these changes are made in a given practice or medical center, the expected impact from each intervention may be inferred from the above-quoted studies, though differences in the patient populations may partly limit external validity.
